# Commentary: Extensional Versus Intuitive Reasoning: The Conjunction Fallacy in Probability Judgment

**DOI:** 10.3389/fpsyg.2015.01832

**Published:** 2015-11-25

**Authors:** Peter Lewinski

**Affiliations:** Faculty of Economics, Université de NeuchâtelNeuchâtel, Switzerland

**Keywords:** conjunction fallacy, linda problem, probability judgment, intuitive reasoning, bounded rationality

The “Linda problem” (Tversky and Kahneman, 1983) is arguably one of the best-known examples of how people commit the conjunction fallacy (cited 1100 times in Web of Science as of November 2015). In this broadly recognized experiment, more than 80 percent of participants failed to recognize the conjunction rule, one of the most fundamental statistical laws, which expresses that the probability of both “A” and “B” being true cannot be higher than the probability of “A” alone being true.

This commentary provides possible alternative accounts of the conjunction fallacy in the Linda problem in light of bounded rationality theory and “nudging” (or “libertarian paternalism,” Gigerenzer, 2015) and generates novel hypotheses in the domain of experimental psychology.

Tversky and Kahneman (1983) have long argued that humans do not reason rationally and are subject to many “cognitive illusions,” such as overconfidence bias, contingency illusions or the false consensus effect. One such cognitive illusion is the conjunction fallacy from the Linda problem. However, this view has been challenged; for instance, Gigerenzer and his colleague (Hertwig and Gigerenzer, 1999) claim that this conjunction “error” may result from the human capacity for inferring additional semantic information from social situations and not from “flaws” in human cognition, as Tversky and Kahneman would argue.

We argue that usually, at the initial encounter between two strangers, more rather than less information about the other person is preferable, as a means to better manage self-image and know how to behave and how to present oneself. Given these premises, two pieces of information as opposed to one piece can be preferred in the Linda problem. Therefore, if one can establish two details about a person instead of only one, the more encompassing description of a person should be chosen more frequently. Imagining a version of Linda in which she is a bank teller and quite probably an active feminist, we think, is presumably easier than imagining that she is only a bank teller—though with many other unaccounted for or “left-over” attributes, such as older than 30, outspoken, a participant in demonstrations, etc., (also see original description of Linda in Tversky and Kahneman, 1983).

Furthermore, there seemingly have been no studies checking whether the word “active” contributes to people's preference for the presentation of Linda as a bank teller and active in the feminist movement. After all, her description perfectly fits someone being active in some kind of movement, not necessarily that she is active specifically in a feminist movement. When asked to imagine a meeting with the Linda described in the Linda problem, perhaps many people would visualize a bright, mature woman who is an active participant in some undefined social movement, based solely on her mentioned involvement in various demonstrations.

Another argument in defense of purposely violating the statistical rule of conjunction is the speed and frugality of heuristics. For most people, committing the conjunction fallacy in the semantically rich environment of the Linda problem may be inadvertently preferable to a choice based on purely logical reasoning. Assessing one's “identity” in a manner that is both quickly produced and quickly retrieved later could hypothetically be an adaptive strategy for the contemporary human environment, as opposed to obeying laws of statistics through elaborate, lengthy descriptions.

Yet another experiment likely to produce useful, interesting results would consist of asking participants to depict Linda in one, two, three, four or more adjectives that were not explicitly mentioned in the original description (e.g., being a bank teller, an active feminist, being a wife) and inform them that a more compressed summary is better. If the experiment reveals a preference for details being compressed into more than one word, then it might be argued that people opt to have a more complex image of a person, rather than singular attributes. Such a result would partly explain why choosing the description of Linda as bank teller and active feminist is more preferred than the description of Linda as merely a bank teller.

Critically, many others (e.g., Moro, 2009; Tentori and Crupi, 2012) argued that the conjunction fallacy is a genuine error of *reasoning bias* and not a mere *misunderstanding*. For example, research showed that people are willing to choose conjunctive bets over more likely bets, even though transparent presentations conditions are assured (e.g., Bonini et al., 2004; Nilsson and Andersson, 2010). Still, recent evidence offers an alternative account—such biases might have been instilled and cued in bettors through bookmakers' common strategy of almost solely advertising such special conjunctive bets, resulting in the representativeness heuristic in bettors (see Newall, 2015). To conclude—if a choice must be made, perhaps it is better to consider people as influenced by environmental “nudges” rather than as biased and unreasonable beings *per se*.

Our conclusion is that people supposedly exhibit a substantial amount of flawed reasoning, such as the conjunction fallacy. This is true. However, what is also likely true is that this flawed reasoning is not only possibly “inherent” or that we are biased because our cognitive capacities are limited, but because there are many environmental factors influencing those cognitive capacities. For example, different characteristics of learning environments can cue specific motivational states, which in turn affect learning outcomes (see Lewinski, 2015a for a review). A similar phenomenon occurs in emotion research where automated facial coding software (Lewinski et al., 2014) can outperform people in recognizing neutral faces as neutral because people are socialized into seeing facial emotions that are not there, whereas software is not (Lewinski, 2015b). Therefore, we might be “nudged” to commit those fallacies, including conjunction fallacy, such as bookmakers bombarding their clients with bets of low outcome probability or big multinationals spending billions of dollars to make sure people commit fallacies (e.g., see Gigerenzer, 2015). Conjunction fallacy is irrational, but the size of this irrationality could be smaller if people's environmental and social reality was constructed in such a way that it diminishes those fallacies (and people approve of that if choice architecture is designed toward specifically benefiting individuals and society; Junghans et al., 2015).

## Conflict of interest statement

The author declares that the research was conducted in the absence of any commercial or financial relationships that could be construed as a potential conflict of interest.

## References

[B1] Bonini N. Tentori K. Osherson D. (2004). A different conjunction fallacy. Mind Lang. 19, 199–210. 10.1111/j.1468-0017.2004.00254.x

[B2] Gigerenzer G. (2015). On the supposed evidence for libertarian paternalism. Rev. Philos. Psychol. 6, 361–383. 10.1007/s13164-015-0248-1 26213590 PMC4512281

[B3] Hertwig R. Gigerenzer G. (1999). The ‘conjunction fallacy’ revisited: how intelligent inferences look like reasoning errors. J. Behav. Decis. Mak. 12, 275–305.

[B4] Junghans A. F. Cheung T. T. De Ridder D. D. (2015). Under consumers' scrutiny-an investigation into consumers' attitudes and concerns about nudging in the realm of health behavior. BMC Public Health 15:336. 10.1186/s12889-015-1691-8 25881161 PMC4393866

[B5] Lewinski P. (2015a). Effects of classrooms' architecture on academic performance in view of telic vs paratelic motivation: a review. Front. Psychol. 6:746. 10.3389/fpsyg.2015.00746 26089812 PMC4453269

[B6] Lewinski P. (2015b). Automated facial coding software outperforms people in recognizing neutral faces as neutral from standardized datasets. Front. Psychol. 6:1386. 10.3389/fpsyg.2015.01386 26441761 PMC4565996

[B7] Lewinski P. den Uyl T. M. Butler C. (2014). Automated facial coding: validation of basic emotions and FACS AUs in FaceReader. J. Neurosci. Psychol. Econ. 7, 227–236. 10.1037/npe0000028

[B8] Moro R. (2009). On the nature of the conjunction fallacy. Synthese 171, 1–24. 10.1007/s11229-008-9377-8

[B9] Newall P. W. (2015). How bookies make your money. Judgm. Decis. Mak. 10, 225–231. Available online at: http://journal.sjdm.org/14/141026a/jdm141026a.pdf

[B10] Nilsson H. Andersson P. (2010). Making the seemingly impossible appear possible: effects of conjunction fallacies in evaluation of bets on football games. J. Econ. Psychol. 31, 172–180. 10.1016/j.joep.2009.07.003

[B11] Tentori K. Crupi V. (2012). On the conjunction fallacy and the meaning of and, yet again: a reply to Hertwig, Benz, and Krauss (2008). Cognition 122, 123–134. 10.1016/j.cognition.2011.09.002 22079517

[B12] Tversky A. Kahneman D. (1983). Extensional versus intuitive reasoning: the conjunction fallacy in probability judgment. Psychol. Rev. 90, 4 10.1037/0033-295X.90.4.293 PMC4658440 26635712

